# Antibody-drug conjugates combinations in cancer treatment

**DOI:** 10.37349/etat.2024.00243

**Published:** 2024-06-27

**Authors:** Giulia Pretelli, Kleida Mati, Lucia Motta, Anastasios Stathis

**Affiliations:** National Cancer Center/Cancer Hospital, Chinese Academy of Medical Sciences, China; ^1^Department of Medical Oncology, Vall d’Hebron Institute of Oncology (VHIO), 08035 Barcelona, Spain; ^2^Oncology Unit, SALUS Hospital, 1000 Tirana, Albania; ^3^Medical Oncology Unit, Humanitas Istituto Clinico Catanese, 95123 Catania, Italy; ^4^Department of Clinical and Experimental Medicine, University of Catania, 95123 Catania, Italy; ^5^Oncology Institute of Southern Switzerland, EOC, 6500 Bellinzona, Switzerland; ^6^Faculty of Biomedical Sciences, Università della Svizzera Italiana (USI), 6900 Lugano, Switzerland

**Keywords:** Antibody-drug conjugates, cancer treatment, novel anticancer therapies, combination regimens

## Abstract

Antibody-drug conjugates (ADCs) have emerged as a promising class of anticancer agents. Currently, the Food and Drug Administration has granted approval to 12 compounds, with 2 later undergoing withdrawal. Moreover, several other compounds are currently under clinical development at different stages. Despite substantial antitumoral activity observed among different tumor types, adverse events and the development of resistance represent significant challenges in their use. Over the last years, an increasing number of clinical trials have been testing these drugs in different combinations with other anticancer agents, such as traditional chemotherapy, immune checkpoint inhibitors, monoclonal antibodies, and small targeted agents, reporting promising results based on possible synergistic effects and a potential for improved treatment outcomes among different tumor types. Here we will review combinations of ADCs with other antitumor agents aiming at describing the current state of the art and future directions.

## Introduction

Antibody-drug conjugates (ADCs) represent one of the most rapidly expanding classes of anticancer drugs. Over the last few years, several ADCs have been approved as monotherapy for cancer treatment (Table 1 and 2) and many others are currently in clinical development [1].

**Table 1:** The first approval of ADCs as a single agent by the Food and Drug Administration (FDA) and/or European Medicines Agency (EMA)

**Drug**	**Target**	**Indication**	**FDA/EMA**	**Approval year**	**Sponsor**
Gemtuzumab ozogamicin	CD33	AML	y/y	2000; 2017	Pfizer/Wyeth
Brentuximab vedotin	CD30	HL	y/y	2011	Seattle Genetics
Trastuzumab emtansine	HER2	HER2-positive mBC	y/y	2013	Genetech/Roche
Inotuzumab ozogamicin	CD22	Acute lymphoblastic leukemia	y/y	2017	Pfizer/Wyeth
Moxetumomab pasudotox	CD22	HCL	y/y–withdrawn	2018–withdrawn in July 2023	AstraZeneca
Enfortumab vedotin	Nectin-4	Urothelial cancer	y/y	2019	Astellas/Seattle Genetics
Trastuzumab deruxtecan	HER2	HER2-positive mBC	y/y	2019	AstraZeneca/Daiichi Sankyo
Sacituzumab govitecan	Trop-2	Metastatic TNBC	y/y	2020	Immunomedics
Belantamab mafodotin	BCMA	Multiple myeloma	y/y–withdraw process	2020–withdraw process in November 2022	GlaxoSmithKline
Loncastuximab tesirine	CD19	DLBCL	y/y	2021	ADC Therapeutics
Tisotumab vedotin-tftv	Tissue factor	Recurrent or metastatic cervical cancer	y/n	2021	Seagen Inc
Mirvetuximab soravtansine	FRα	FRα-positive, platinum-resistant epithelial ovarian, fallopian tube, or peritoneal cancer	y/y	2022	ImmunoGen

ADCs: antibody-drug conjugates; AML: acute myeloid leukemia; HCL: hairy-cell leukemia; HL: Hodgkin lymphoma; HER2: human epidermal growth factor receptor 2; mBC: metastatic breast cancer; Nectin-4: nectin cell adhesion molecule 4; Trop-2: trophoblast cell surface antigen-2; TNBC: triple-negative breast cancer; BCMA: B cell maturation antigen; DLBCL: diffuse large B-cell lymphoma; FRα: folate receptor alpha; n: not; y: yes

**Table 2:** First approval of ADCs by other regulatory agencies

**Drug**	**Target**	**Indication**	**Country**	**Approval year**	**Sponsor**
Cetuximab sarotalocan	EGFR	Head and neck cancer	Japan	2020	RakutenMedical
Disitamab vedotin	HER2	HER2^+^ gastric carcinoma	China	2021	RemeGen

ADCs: antibody-drug conjugates; EGFR: epidermal growth factor receptor; HER2: human EGFR 2

**Figure 1:**
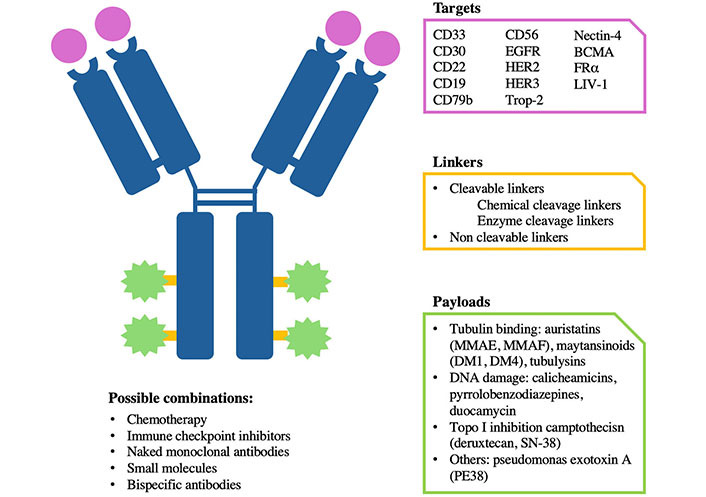
Antibody-drug conjugate (ADC) specificity. Graphic representation of the different components and specificity of an ADC. Each ADC is different from the others depending on the target, payloads, and linker. It can be combined with different drugs. BCMA: B cell maturation antigen; EGFR: epidermal growth factor receptor; HER2: human EGFR 2; FRα: folate receptor alpha; Trop-2: trophoblast cell surface antigen-2; MMAE: monomethyl auristatin-E; MMAF: monomethyl auristatin-F

ADCs consist of three main components: a monoclonal antibody (mAb), a linker, and a cytotoxic drug (also known as the payload) (Figure 1). The payload is connected to the mAb through the linker. Once the mAb component binds to its target antigen, the complex antigen-ADC is internalized in the tumor cell, and the payload is delivered and released at the tumor site. The linker is presently classified as cleavable and non-cleavable. Among cleavable linkers, there are varying degrees of stability: linkers less stable may trigger the bystander effect when cleaved, releasing the payload far from the targeted tumor cells and causing the destruction of neighboring cells. Non-cleavable linkers are stable in circulation and release the payload after internalization in response to lysosomal enzymes. Considering that the bystander effect is recognized as a significant component of ADC activity, optimizing linker stability is crucial for ADC effectiveness. The proportions of the three components circulating in the bloodstream differ based on the type of linker used and the overall integrity of the molecule [2].

Despite the initial activity, tumor cells eventually develop resistance to ADCs limiting their use [3, 4]. Several mechanisms of resistance have been described, including changes at the antigen level (such as altered expression or mutations), changes in endocytosis mechanisms and vesicular trafficking, defects of lysosomal activity (pH, proteolytic enzymes), imbalance in proapoptotic and antiapoptotic factors, alteration of signaling pathways, increased activity of the drug efflux pumps [3, 4]. To overcome resistance several strategies are being actively investigated including improvements of the ADC components such as modifications of the cytotoxic agent, in order to reduce the affinity of efflux pumps, modifications of the linker, use of bispecific or biparatopic ADCs, and the development of combination strategies [3].

Combination strategies with other anticancer agents have been regarded as a potential approach to enhance the efficacy of ADCs and overcome resistance, ultimately improving treatment outcomes [3–5]. The ideal combination should be with a drug that contributes to the antitumor effect in a synergistic way, while it has minimal overlapping toxicities [6].

As of today, combinations of ADCs with other drugs have been approved mainly in the field of hematologic malignancies (Table 3). Among them, brentuximab vedotin (BV) and polatuzumab vedotin (PV), have been developed in combinations with chemotherapeutic agents and with rituximab for the treatment of various types of B-cell and T-cell lymphoma [7–10]. Additionally, gemtuzumab ozogamicin (GO) in combination with chemotherapy has been approved for the treatment of acute myeloid leukemia (AML) [11]. More recently, the combination of enfortumab vedotin (EV) and pembrolizumab has received Food and Drug Administration (FDA) approval for treating locally advanced or metastatic urothelial carcinoma (la/mUC), a combination that demonstrated improved overall survival (OS) compared to the standard of care [12].

**Table 3:** FDA and/or EMA-approved combinations of ADCs

**Combination**	**Approval year**	**Patient population**	**Phase**	**Primary endpoint**	**Comparator arm**	**Primary endpoint results**	**G3–4 AEs**
Gemtuzumab ozogamicin + daunorubicin + cytarabine	2017	Untreated AML; R/R AML	III	EFS	DA	EFS 40.8% *vs.* 17.1%	13% *vs.* 9.2%
Gemtuzumab ozogamicin + induction CT and intensification CT	2020	Untreated AML pediatric patient	III	EFS	Induction CT and intensification CT	EFS 48% *vs.* 40.0%	76% *vs.* 76%
Brentuximab vedotin + AVD	2018	Untreated HL	III	PFS	ABVD	PFS 82.1% *vs.* 78.8%	83% *vs.* 66%
Brentuximab vedotin + CHP	2018	Untreated PTCL/ sALCL	III	PFS	CHOP	PFS 57.1% *vs.* 44.4%	66% *vs.* 65%
Brentuximab vedotin + AVEPC	2022	Pediatric untreated HL	III	EFS	ABVE-PC	NR	73.5% *vs.* 68.2
Polatuzumab vedotin + BR	2019	R/R DLBCL	Ib–II	Safety, tolerability, CR rate	BR	CR 40.0% *vs.* 17.5%	Anemia 28.2% *vs.* 17.9%, Neutropenia 46.2% *vs.* 33.3%
Polatuzumab vedotin + R-CHP	2023	Untreated DLBCL, NOS or HGBL	III	PFS	R-CHOP	PFS 76.7% *vs.* 70.2%	60.7% *vs.* 69.8%
Enfortumab vedotin + Pembro	2023	Untreated la/mUC	III	PFS/OS	Gemcitabine + Cisplatin/Carboplatin	PFS 12.5% *vs.* 6.3% OS 31.5% *vs.* 16.1%	55.9% *vs.* 69.5%

FAD: Food and Drug Administration; EMA: European Medicines Agency; ADCs: antibody-drug conjugates; G3–4: grade 3–4; AEs: adverse events; R/R: relapsed or refractory; AML: acute myeloid leukemia; EFS: event-free survival; DA: daunorubicin and cytarabine; CT: chemotherapy; AVD: doxorubicin-vinblastine-dacarbazine; PFS: progression-free survival; ABVD: doxorubicin-bleomycin-vinblastine-dacarbazine; PTCL: peripheral T-cell lymphoma; sALCL: systemic anaplastic large-cell lymphoma; CHP: cyclophosphamide-doxorubicin-prednisone; CHOP: cyclophosphamide-doxorubicin-vincristine-prednisone; CR: complete response; HL: Hodgkin lymphoma; ABVE-PC: doxorubicin-bleomycin-vincristine-etoposide-prednisone-cyclophosphamide; NR: not reached; BR: bendamustine-rituximab; DLBCL: diffuse large B-cell lymphoma; NOS: not otherwise specified; HGBL: high-grade B-cell lymphoma; R-CHOP: rituximab-CHOP; R-CHP: rituximab-CHP; OS: overall survival; AVEPC: doxorubicin-vincristine-etoposide-prednisone-cyclophosphamide; la/mUC: locally advanced or metastatic urothelial carcinoma; Pembro: pembrolizumab

Despite these approvals, there is still a lot of space for improvement, and many are ongoing trials evaluating ADCs in combinations with various anti-cancer agents. Among the most studied combinations, those with chemotherapy face often the challenge of toxicities, which may depend on off-target toxicities but also on characteristics of the ADC such as the presence of cleavable linkers and high drug-to-antibody ratio [6]. On the other hand, despite many clinical trials evaluating combinations with immune checkpoint inhibitors (ICIs), only the combination mentioned above of EV with pembrolizumab demonstrated an OS benefit compared to the previous standard therapy [6, 12]. Finally, the hypothesis that a dual ADC-targeted agent blockade could improve therapeutic efficacy remains intriguing, in particular with the advancement of new-generation ADCs [6]. Here, we will review the main clinical results of combinations of ADCs with other anti-cancer drugs.

## Combinations in clinical development: ADCs combined with chemotherapy

The synergy between the cytotoxic payload delivered by the ADC and the chemotherapeutic agent arises from the dual cytotoxic impact on the tumor cells, similar to traditional chemotherapy combinations using drugs with distinct mechanisms of action. This approach aims at preventing the development of resistance typically associated with single-agent therapy. However, combining ADCs with cytotoxic agents poses challenges, primarily due to the risk of overlapping toxicities. The ideal chemotherapeutic partner should thus have the ability to enhance effectiveness and not increase toxicities. A deeper understanding of the cell cycle and the phases in which the different agents act and knowledge of how chemotherapy influences the modulation of surface antigen expression may help in deciding the combinations to develop [6]. Numerous combinations of ADCs with cytotoxic agents are in development for both solid tumors and hematologic malignancies. Certain combinations have already received approval for treating lymphoma and leukemia (Table 3). In the following paragraphs, we will outline the main clinical findings of combinations of ADCs with chemotherapy (Table S1).

### ADCs approved as single agents combined with chemotherapy: main results of clinical trials

#### Gemtuzumab ozogamicin

GO is a recombinant mAb targeting the CD33 antigen, conjugated via a cleavable linker to a cytotoxic antibiotic derivative of calicheamicin [13]. In May 2000, the FDA granted accelerated approval to GO for patients with CD33-positive relapsed AML who were not suitable for conventional chemotherapy [13, 14]. However, the phase III trial SWOG S0106 found no statistically significant difference in outcomes from the addition of GO to chemotherapy [daunorubicin and cytarabine (DA)] compared to chemotherapy alone, with a higher mortality rate in the combination arm. Based on these results, in June 2010, Pfizer voluntarily withdrew GO from the market [15]. Nonetheless, GO was further evaluated with DA using alternative fractionated dosing schedules in the phase III ALFA-0701 trial and as monotherapy in the phase II AML-19 trial [16, 17]. Administration of lower fractionated doses of GO in combination with chemotherapy in the ALFA-0701 trials resulted in significant improvements in event-free survival (EFS) and OS, although with a higher frequency of grade 3 (G3) or higher adverse events (AEs) in the GO group (predominantly infections and skin toxicities) [16]. Based on these results, GO was re-approved by the FDA in 2017 with DA or as a monotherapy for the treatment of patients with CD33-positive newly-diagnosed AML. In June 2020 the approval was extended to the pediatric population based on the results of the AAML0531 trial, which demonstrated better outcome for patients receiving the combination of GO and chemotherapy compared to chemotherapy alone [18]. Other studies have explored the combination of GO and other chemotherapy regimens in AML patients, such as cytarabine and mitoxantrone [19] and high-dose cytarabine, mitoxantrone, and all-trans retinoic acid [20]. Additionally, ongoing research is exploring the association of GO, mitoxantrone and etoposide (NCT03839446) and a liposomal cytarabine-daunorubicin (NCT05558124) for the same patient population.

#### Brentuximab vedotin

BV is an anti-CD30 mAb conjugated through a protease-cleavable linker to the anti-mitotic cytotoxic agent monomethyl auristatin-E (MMAE) [21]. Based on its demonstrated clinical efficacy and its approval for treating patients with Hodgkin lymphoma (HL) and anaplastic large cell lymphoma [7–9], BV was further investigated in combination with other treatments. In a phase I trial involving treatment-naive HL patients, BV was assessed in combination with the standard ABVD (doxorubicin-bleomycin-vinblastine-dacarbazine) regimen or the modified AVD (ABVD without bleomycin) regimen [22]. Results showed a comparable rate of complete response (CR) but a significantly higher rate of G3 AEs, in particular pulmonary toxicities, in the ABVD arm. Based on these results, it was concluded that BV should not be used in bleomycin-containing regimens like ABVD [22]. The phase III ECHELON-1 study compared BV plus AVD to the standard ABVD regimen in patients with previously untreated stage III or IV HL. The experimental arm resulted in improved progression-free survival (PFS) and OS rates compared to the ABVD group [23, 24], with a manageable safety profile. Based on these results, in March 2018 the FDA approved the combination of BV with AVD for the treatment of previously untreated stage III/IV HL [25]. The superiority of BV plus chemotherapy, compared to chemotherapy alone, was demonstrated also in patients with previously untreated peripheral T-cell lymphoma (PTCL). In phase III ECHELON-2 trial, the standard CHOP (cyclophosphamide-doxorubicin-vincristine-prednisone) regimen was compared to the experimental arm of BV with CHP (a modified CHOP with the omission of vincristine due to overlapping neurotoxicity with BV), showing an improvement in both PFS and OS in the experimental arm, with a similar rate of G3 or higher AEs [26, 27]. This combination has been approved by the FDA in November 2018. In November 2022, a significant advancement in treatment emerged when the FDA approved a third combination involving brentuximab with chemotherapy. This approval represents a notable addition to the available therapeutic options for pediatric patients aged 2 years and older with previously untreated high-risk classical HL (cHL). The phase III study AHOD1331 demonstrated superior outcomes with the combination of BV alongside doxorubicin, vincristine, etoposide, prednisone, and cyclophosphamide in comparison to the standard ABVE-PC (doxorubicin-bleomycin-vincristine-etoposide-prednisone-cyclophosphamide) arm [28]. Numerous other trials are currently ongoing, exploring combinations with different chemotherapeutic regimens in hematologic malignancies A large phase III trial including 1,500 patients has compared the remodeled combination regimen BrECADD (BV, added to etoposide, cyclophosphamide, doxorubicin, dacarbazine, dexamethasone) versus the escalated BEACOPP (bleomycin-etoposide-doxorubicin-cyclophosphamide-vincristine-procarbazine-prednisone) regimen in patients with newly diagnosed advanced risk HL. Preliminary results showed no inferiority of the new regimen [29].

#### Polatuzumab vedotin

PV consists of an anti-CD79b mAb conjugated to MMAE through a cleavable linker [10]. PV, which was not approved as a single agent, in June 2019 received FDA approval for the treatment of patients with relapsed or refractory (R/R) diffuse large B-cell lymphoma (DLBCL) in combination with bendamustine-rituximab (BR) based on the results of a phase Ib/II trial that compared its efficacy and safety to bendamustine and rituximab [30]. Among other combinations with chemotherapy in different lymphoma subtypes, the most significant has been the phase III POLARIX trial that compared the combination with rituximab-CHP (R-CHP) to the standard rituximab-CHOP (R-CHOP) regimen in patients with untreated DLBCL. The experimental arm resulted in PFS benefits with similar OS rates [31]. This study led to the FDA approval of the combination PV-R-CHP in April 2023 for the treatment of untreated DLBCL, not otherwise specified (NOS), or high-grade B-cell lymphoma (HGBL).

#### Inotuzumab ozogamicin

Inotuzumab ozogamicin (INO), an anti-CD22 antibody conjugated to a calicheamicin payload via a cleavable linker [32], received FDA approval for use as a single agent in patients with R/R B-cell precursor acute lymphoblastic leukemia (ALL), based on the phase III IN-NOVATE trial [32]. A single-arm phase II trial investigated the efficacy of INO with mini-hyper CVD (cyclophosphamide-vincristine-methotrexate-cytarabine), with or without blinatumomab, in patients with B-cell ALL. It showed promising efficacy in terms of OS and even more favorable survival outcomes in the blinatumomab arm [33]. In the subgroup of older patients (≥ 60 years) with Philadelphia chromosome-negative B-cell ALL, more than 70% of the patients experienced G3–4 hematologic toxicity. Consequently, there is a need to further adjust and refine the combination regimen to enhance tolerability [34]. Other studies evaluated the efficacy and safety of INO in combination with various chemotherapy agents.

#### Trastuzumab emtansine

Trastuzumab emtansine (T-DM1) is an ADC that combines the human epidermal growth factor receptor 2 (HER2)-targeting humanized mAb trastuzumab with a maytansinoid toxin-DM1, through a non-cleavable linker [35]. It became the first ADC approved for the treatment of a solid malignancy, based on the phase III EMILIA trial which included patients with advanced breast cancer (BC) and resulted in a benefit in PFS and OS compared to lapatinib plus capecitabine [36, 37]. Following the results of the KATHERINE study, T-DM1 was also approved for patients with HER2-positive BC with residual disease after neoadjuvant therapy [38] and has been evaluated in other HER2-positive solid tumors [39, 40]. Combination therapies of T-DM1 with chemotherapy regimens (such as docetaxel and capecitabine) did not result in any improvements and were associated with increased toxicity [41, 42]. The phase II TRAXHER2 trial evaluated the efficacy and safety of T-DM1 in combination with capecitabine compared to T-DM1 alone in patients with metastatic BC (mBC). Patients in the combination arm experienced a higher rate of G3–4 AEs, without any significant benefit in clinical outcomes [41]. An increased rate of AEs resulting from overlapping toxicities was also demonstrated in two phase Ib/IIa studies that evaluated T-DM1 in combination with docetaxel and paclitaxel, with or without pertuzumab, in patients with mBC or locally advanced BC (LABC). While the combination showed significant clinical activity, its clinical use is limited due to the occurrence of AEs, leading to frequent dose reductions and interruptions [42, 43]. Therefore, there is a need to seek a different partner that could be safely combined with T-DM1. A potential combination with promising preclinical data could involve gemcitabine, which has been shown to upregulate the expression of HER2 in pancreatic ductal adenocarcinoma cells and BC cells [44, 45]. Presently, no ongoing clinical trials are evaluating this combination.

#### Trastuzumab deruxtecan

Trastuzumab deruxtecan (T-DXd) is an ADC consisting of an anti-HER2 mAb and a topoisomerase I inhibitor, the exatecan derivative DXd [46]. T-DXd was approved by the FDA for the treatment of HER2-positive and HER2-low mBC patients [47, 48] and HER2-positive gastric adenocarcinomas [49]. Additionally, the FDA has granted accelerated approval for T-DXd in the treatment of metastatic HER2-mutant non-small cell lung cancer (NSCLC) [50] and breakthrough therapy designations for treating patients with HER2-positive metastatic colorectal cancer (mCRC) and advanced HER2-positive solid tumors [51, 52]. T-DXd combinations are currently being investigated in ongoing clinical trials. The phase I/IIb Destiny-Breast07 trial is exploring various regimens, including combinations of T-DXd with paclitaxel for patients with HER2-positive mBC [53]. Additionally, another phase Ib study, Destiny-Breast08, will assess five different regimens, incorporating combinations of T-DXd with capecitabine, anastrozole, and fulvestrant in HER2-low mBC patients [54]. The investigation of treatment combinations involving T-DXd and chemotherapy extends beyond BC. In advanced HER2-positive gastric cancer, the phase Ib/II Destiny-Gastric03 trial is currently assessing T-DXd in combination with cytotoxic chemotherapy agents [5-fluorouracil (5-FU), capecitabine, oxaliplatin] and/or immunotherapy agents [55].

#### Mirvetuximab soravtansine

Mirvetuximab soravtansine (MIRV) is an ADC consisting of a humanized folate receptor alpha (FRα)-targeting mAb connected to the maytansinoid DM4, which induced mitotic arrest by suppressing microtubule dynamics [56]. Based on the results of the phase III SORAYA trial, the FDA granted MIRV priority review and subsequently accelerated approval in November 2022 for patients with platinum-resistant epithelial ovarian cancer expressing FRα [57]. MIRV has been studied in a phase Ib trial in platinum-sensitive, relapsed ovarian cancer patients in combination with carboplatin. The combination demonstrated clinical activity and a manageable safety profile [58].

### Combinations of not-approved ADCs with chemotherapy

#### Anetumab ravtansine

Anetumab ravtansine (AR), an ADC composed of a fully human IgG1 anti-mesothelin mAb linked to the tubulin inhibitor DM4 via a cleavable linker, has demonstrated high cytotoxic activity in preclinical studies against mesothelin-expressing tumors such as mesothelioma, pancreatic cancer, NSCLC, and ovarian cancer [59]. Encouraging clinical activity was demonstrated in patients with advanced or metastatic solid tumors, particularly in mesothelioma patients [60]. Results from a phase Ib trial showed that the combination with pegylated-liposomal doxorubicin exhibited clinical activity and tolerability in patients with platinum-resistant ovarian cancer [61].

#### Depatuxizumab mafodotin

Depatuxizumab mafodotin (Depatux-M) is an ADC that targets the epidermal growth factor receptor (EGFR). It consists of the humanized recombinant mAb ABT-806, which is linked via a non-cleavable linker to the anti-microtubule agent monomethyl auristatin-F (MMAF) [62]. In the phase II trial INTELLANCE 2, the combination of Depatux-M and temozolomide (TMZ) was investigated in patients with recurrent EGFR-amplified glioblastoma. This study compared Depatux-M alone or in combination with TMZ versus lomustine or TMZ [63]. The combination arm showed improved OS compared to the control arm, suggesting a potential clinical benefit. The most common AE in the Depatux-M arms was reversible corneal epitheliopathy G3–4 [63]. A multicenter study conducted by the Italian Association of Neuro-Oncology further investigated this combination treatment in patients with recurrent glioblastoma and reported similar results. However, larger prospective studies would be necessary to confirm its efficacy and further explore its safety [64]. There are currently no ongoing studies.

#### Lorvotuzumab mertansine

Lorvotuzumab mertansine (LM) is a humanized anti-CD56 mAb linked via a cleavable linker to the maytansinoid DM1 [65]. It was evaluated in a phase I/II trial in combination with carboplatin and etoposide, in comparison to carboplatin and etoposide alone. This study involved patients with untreated extensive-stage small-cell lung cancer but yielded disappointing results both in terms of safety and efficacy [66]. The drug is no longer being developed, there are no ongoing studies with LM.

## Combinations in clinical development: ADCs combined with ICIs

The rationale for the development of combinations with ICIs lies in their complementary immunomodulatory effects. ADCs target specific tumor antigens and may enhance tumor antigen presentation and T-cell infiltration an effect that can be complemented by ICIs [67]. Numerous ADC combinations with ICIs have been explored in preclinical and early clinical studies. The recent FDA approval of EV in combination with pembrolizumab for patients with la/mUC marks a significant milestone in the development of new combinations [12]. Here we will review clinical trials evaluating combination therapies of ADCs with ICIs (Table S2).

### ADCs approved as single agents combined with ICI: main results of clinical trials

#### Enfortumab vedotin

EV is an ADC directed against Nectin-4 and comprises a fully human mAb linked to MMAE [6]. It demonstrated survival benefits as monotherapy in pretreated patients with la/mUC [6] and received FDA approval in December 2023 in combination with pembrolizumab [5]. The effectiveness of the combination relies on EV’s ability to trigger immunogenic cell death and boost the infiltration of T-cells. Pembrolizumab further enhances the anti-tumor immune response, complementing EV’s actions [7]. The approval was based on the results of the EV-302/KN-A39 trial which demonstrated significant improvements in PFS and OS for patients with la/mUC treated with EV and pembrolizumab compared to platinum-based chemotherapy, confirming EV with pembrolizumab as the new standard of care for first-line la/mUC [5]. Further expanding the clinical exploration of EV combinations, the VOLGA trial (NCT04960709) assesses its combination with durvalumab and tremelimumab in neoadjuvant and adjuvant settings in patients with muscle-invasive BC (MIBC). This trial targets a patient population ineligible for cisplatin-based chemotherapy, addressing a significant unmet need in MIBC management. The rationale relies on using EV’s capability to induce immunogenic cell death in conjunction with the immune-modulating effects of two ICIs. This aims to improve disease control before surgery and delay the recurrence [8].

#### Brentuximab vedotin

The combination of BV and ICIs has been a focus of several clinical trials. The phase I/II trial, CheckMate 436, evaluated the combination of BV and nivolumab in patients with R/R primary mediastinal B-cell lymphoma (PMBL). This trial showed significant anti-tumor activity and a manageable safety profile, emphasizing the efficacy and safety of BV with nivolumab [68]. Furthermore, BV was also examined in another phase I/II study involving patients with R/R HL in combination with ipilimumab, nivolumab, or both. These combinations demonstrated high activity and maintained generally favorable safety profiles, with follow-up reports indicating benefits in PFS [9, 69, 70]. Currently, ongoing phase II and phase III trials (NCT04561206, NCT03138499) aim to further assess the combination of nivolumab with BV. Additionally, a small cohort study involving BV and pembrolizumab was conducted as a single-center retrospective analysis on 10 patients with R/R HL. The study revealed impressive results in objective response rate (ORR) and complete metabolic response rate, along with a rapid median time to best response [71]. An ongoing phase II clinical trial (NCT04609566) is set to evaluate the efficacy and safety of this combination in patients with metastatic solid tumors after progression on prior programmed cell death 1 (PD-1) inhibitors [72]. Other studies are also underway, assessing the combination of BV and pembrolizumab in R/R HL, R/R T-cell lymphoma, and recurrent PTCL (NCT05180097, NCT05313243, NCT04795869).

#### Trastuzumab emtansine

Based on evidence suggesting that T-DM1 could elicit antitumor immunity and render the tumor cells sensitive to ICIs [73], the drug has been evaluated in combination with atezolizumab and pembrolizumab in various clinical trials. The phase 2 KATE2 trial evaluated T-DM1 with atezolizumab in patients with previously treated HER2-positive advanced BC. Although it did not show a significant improvement in PFS for the overall population, subgroup analysis indicated a PFS advantage for patients with programmed cell death ligand 1 (PD-L1) positive tumors [74]. These findings have led to the initiation of the phase III KATE3 trial (NCT04740918), focusing on patients with HER2-positive and PD-L1-positive LABC/mBC [75]. Furthermore, a phase Ib trial investigated atezolizumab with T-DM1 in HER2-positive early BC (eBC), LABC, or mBC, showing an acceptable safety profile, along with an enhanced adaptive immune response in eBC tumors compared to those with mBC [76]. In another phase I study, investigating the combination of T-DM1 and pembrolizumab in patients with HER2-positive mBC, the regimen exhibited clinical activity and was well tolerated. However, biomarker analyses were constrained due to the small sample size of the cohort, highlighting the need for larger studies to determine predictive markers of response [77, 78].

#### Trastuzumab deruxtecan

Following the results of the phase II DESTINY-Breast01 and phase 3 DESTINY-Breast04 trials in BC patients, along with data from preclinical models [79] new combination strategies are being investigated, incorporating T-DXd and ICIs in HER2-expressing tumors. A phase Ib study assessed T-DXd in combination with nivolumab for HER2-expressing advanced breast or urothelial cancers. This study reported promising results, with a disease control rate (DCR) of 90.6% in HER2-positive patients, and 75% in those with HER2-low BC, an acceptable safety profile, and a benefit in PFS [80]. The phase Ib/II BEGONIA trial delved deeper into T-DXd, this time combining it with durvalumab for untreated HER2-low expressing triple-negative BC (TNBC). Preliminary results were impressive, demonstrating a 100% ORR. Further data is anticipated to elucidate the impact of PD-L1 expression on these outcomes [81]. Additionally, the combination of T-DXd with pembrolizumab is currently under investigation in an ongoing phase Ib trial, targeting patients with HER2-expressing advanced/mBC or NSCLC [82]. Another study in the pipeline is the phase Ib/II trial in gastric cancer, DESTINY-Gastric03 (NCT04379596). In addition, ongoing clinical trials are evaluating the safety and antitumor activity of T-DXd, durvalumab, and pertuzumab for HER2-positive mBC (NCT04538742, NCT04784715).

#### Sacituzumab govitecan

Sacituzumab govitecan (SG) is composed of the mAb anti-Trop2 linked to the active metabolite of irinotecan, SN-38 [83, 84]. The drug is FDA-approved as a single-agent treatment for breast and urothelial cancer. The TROPHY-U-01 Cohort 3 evaluated the combination of SG with pembrolizumab in patients with metastatic urothelial cancer who progressed after platinum-based regimens, showing an encouraging ORR and clinical benefit rate, as well as a manageable safety profile [85]. In addition to this, ongoing research is evaluating the activity of SG in various other clinical contexts and at earlier stages of treatment. For instance, the EVOKE-02 phase II trial is assessing SG in combination with chemotherapy and ICIs as a first-line treatment for patients with non-oncogene addicted NSCLC (NCT05186974). Similarly, a phase I/II study explored the potential of SG when combined with ipilimumab and nivolumab as a first-line therapy for cisplatin-ineligible advanced urothelial carcinoma (UC), demonstrating antitumor activity for this patient population representing an unmet medical need [86].

#### Tisotumab vedotin

Based on the findings from the innovaTV 204 trial, the FDA granted accelerated approval to tisotumab vedotin (TV), which targets tissue factors and is linked to MMAE, for patients with recurrent or metastatic cervical cancer (r/mCC) [87]. The dose expansion arms of the phase Ib/II trial innovaTV 205/GOG-3024/ENGOT-cx8, evaluated TV with carboplatin as first-line treatment or with pembrolizumab as first or second-/third-line treatment in patients with r/mCC. The study met its primary endpoint demonstrating promising anti-tumor activity and acceptable safety profiles [88].

#### Mirvetuximab soravtansine

Preclinical data suggest that MIRV may activate monocytes and upregulate immunogenic cell death markers in ovarian cancer cells [89]. Building on these findings, the phase Ib/II FORWARD II study delved further into the potential of MIRV, in combination with pembrolizumab and bevacizumab, focusing on patients with platinum-resistant ovarian cancer. The combination of MIRV with pembrolizumab was generally well tolerated, with few G3 AEs [90]. Complementing these findings, additional research is being conducted in patients with endometrial cancer (NCT03835819).

### Combination of not-approved ADCs with ICIs

#### Ladiratuzumab vedotin

Ladiratuzumab vedotin (LV) is a novel ADC that combines anti-LIV-1 mAb with MMAE via a protease-cleavable linker [91]. LIV-1 is a transmembrane protein with zinc transporter and metalloproteinase activity, primarily expressed in melanoma, breast, and prostate cancers, while having limited expression in normal tissues [91]. Early-phase studies have shown promising antitumor activity, particularly in heavily treated metastatic TNBC [92]. The combination of LV with pembrolizumab has been evaluated in the first-line therapy of patients with TNBC demonstrating a good tolerability profile and clinical activity [93]. Ongoing research is currently exploring LV in combination with atezolizumab for locally advanced and metastatic TNBC (NCT03424005).

#### Disitamab vedotin

Disitamab vedotin (RC48-ADC) is an anti-HER2 ADC composed of a novel anti-HER2 mAb (hertuzumab), coupled with MMAE by a cleavable linker [94]. Promising data have been observed in both HER2-positive and HER2-negative populations with la/mUC [95]. In a phase Ib/II trial RC48-ADC was studied in combination with toripalimab, an anti-PD-1 antibody known for its clinical activity in UC [96, 97]. The combination showed an ORR of 75% in patients with la/mUC. The ORR was even higher for patients who were HER2 positive and PD-L1 positive. However, antitumor activity was also observed in patients with HER2 2+, 1+, 0, and in those with a PD-L1 level below 1 [96]. The same combination was explored in patients with HER2-expressing advanced gastric or gastroesophageal junction with similar, positive, findings [98].

#### Anetumab ravtansine

A study with AR combined with pembrolizumab in pleural mesothelioma patients showed a higher stable disease rate and median PFS than pembrolizumab alone, although these weren’t statistically significant, possibly due to a smaller sample size [99]. Furthermore, a phase Ib study in pancreatic cancer showed a good DCR and tolerability for AR combined with immunotherapy or chemotherapy [100].

#### Belantamab mafodotin

Belantamab mafodotin (BM) is a novel ADC developed using a B cell maturation antigen (BCMA)-targeted mAb. BCMA, a part of the tumor necrosis factor (TNF) receptor superfamily, is expressed on both normal and malignant plasma cells, as well as late B-cells [101, 102]. The antibody component is linked to MMAF through a protease-resistant linker [103]. BM’s efficacy in treating R/R multiple myeloma (MM) has been evaluated in several clinical studies, demonstrating benefits in PFS, OS, and a manageable safety profile [104]. These results initially led to its FDA approval for monotherapy in R/R MM patients who had undergone four or more lines of therapy. However, in November 2022, this approval was withdrawn following the outcomes of the DREAMM-III study, which did not meet the FDA’s accelerated approval guidelines (NCT04162210). In experimental studies, combining BM with an OX40 agonist has been shown to enhance anti-cancer effects, resulting in increased activity of T cells and dendritic cells within tumors [105]. Clinical trials such as the DREAMM-5 study are exploring this approach, investigating the combination of BM with various immune therapies, including anti-PD-1 and anti-inducible T-cell co-stimulator (ICOS) antibodies, and a γ-secretase inhibitor [106]. A preliminary analysis of 23 patients in this trial indicated that BM combined with anti-ICOS displayed encouraging clinical activity and a manageable safety profile through dose modifications [106]. Additionally, the DREAMM-4 study, which investigated the combination of BM and pembrolizumab, concluded that this combination yielded a favorable ORR and had a safety profile comparable to BM monotherapy [107].

#### Datopotamab deruxtecan

Datopotamab deruxtecan (Dato-DXd) is a novel ADC comprising a humanized anti-TROP2 IgG1 mAb linked to a potent DNA topoisomerase I inhibitor via a cleavable linker [108]. Early phase trials from the TROPION series evaluated the efficacy and safety of Dato-DXd in multiple tumors at different stages, revealing promising clinical activity in both NSCLC and TNBC [109]. Encouraging results from early trials have led to further exploration of Dato-DXd in combination with ICIs. For instance, The TROPION-Lung02 trial investigated Dato-DXd with pembrolizumab ± chemotherapy in metastatic NSCLC patients, reporting an acceptable safety profile and clinical activity. This has led to ongoing studies like TROPION-Lung07 and TROPION-Lung08, which aim to explore Dato-DXd in combination with ICIs, with or without chemotherapy, potentially as first-line treatments [110]. In metastatic TNBC, the phase Ib/II BEGONIA trial evaluated the combination of Dato-DXd and durvalumab, demonstrating a highly encouraging ORR of 79% regardless of PD-L1 expression level, with a safety profile consistent with the known profiles of both agents [111]. Additionally, other studies are assessing the same combination in different stages of BC, ranging from perioperative treatment to therapy of advanced disease (NCT06112379, NCT05629585, NCT06103864). Finally, TROPION-PanTumor03 is set to evaluate Dato-DXd both as monotherapy and in combination with other antitumor agents across various solid cancer types (NCT05489211).

## Combinations in clinical development: ADCs combined with targeted therapy (mAbs and small molecules)

Combinations of ADCs with small targeted therapies such as a tyrosine kinase inhibitor (TKI) or others or with mAb hold substantial promise as they may offer increased selectivity, potentially enhancing the therapeutic effectiveness of the treatment. Here we will review clinical trials evaluating combination therapies of ADCs with naked mAbs and small targeted agents (Table S3).

### ADCs approved as single agents combined with targeted therapies: main results of clinical trials

#### Brentuximab vedotin

The ECHELON-3 study evaluated a novel combination therapy of BV, lenalidomide, and rituximab for R/R DLBCL in patients ineligible for hematopoietic stem cell transplantation (HSCT) or CAR-T therapy. The study involved 10 patients, revealing a 70% ORR with a manageable safety profile, indicating the promising efficacy of this triplet regimen in R/R DLBCL, with the randomized study phase currently ongoing [112].

#### Polatuzumab vedotin

PV is currently being evaluated in combination with rituximab and bispecific antibodies [113–116]. The phase Ib/II study combining PV with mosunetuzumab, a bispecific antibody targeting CD20 and CD3, in relapsed/refractory B-cell non-HL demonstrated promising safety and efficacy, especially for elderly patients with limited treatment options [116]. Additionally, a phase II study evaluating rituximab with either PV or pinatuzumab vedotin in a similar patient population showed efficacy, with a preference for rituximab-PV due to longer response duration and a better safety profile [115]. A phase Ib/II study evaluated PV combined with obinutuzumab and lenalidomide in patients with heavily pre-treated refractory follicular lymphoma [69]. Additionally, PV was studied in combination with bcl-2 inhibitor venetoclax, and as part of a triplet therapy with both venetoclax and rituximab [117, 118]. The phase Ib study investigated the combination of PV with venetoclax and rituximab in R/R DLBCL, showing promising activity and a favorable safety profile [117]. The same combination was explored in the patients with R/R follicular lymphoma, replacing rituximab with obinutuzumab, also yielding encouraging results [119, 120].

#### Inotuzumab ozogamicin

The combination of INO and rituximab was explored in a phase I/II trial in patients with DLBCL of follicular lymphoma, showing high antitumor activity and a manageable safety profile [121]. A phase III trial failed to demonstrate the superiority of the experimental arm compared to the standard [122]. A phase I trial evaluated the combination of INO with temsirolimus in patients with R/R CD22-positive B-cell non-HL. Due to the high rate of toxicities at therapeutic doses, it was concluded that further development of this drug combination was not feasible, despite demonstrating clinical activity [123]. Another early-phase trial explored the combination of INO with bosutinib for R/R Philadelphia chromosome-positive ALL or the lymphoid blast phase of chronic myeloid leukemia, demonstrating clinical activity in terms of ORR and a good tolerability profile [124].

#### Loncastuximab tesirine

Loncastuximab tesirine (LT) is an anti-CD19 ADC, linked to a pyrrolobenzodiazepine dimer cytotoxin, SG3199 [125]. The results of the LOTIS-2 trial led to the FDA’s approval of LT as a single agent for treating patients with R/R large B-cell lymphoma (DLBCL, transformed DLBCL, and HGBL) [125]. Results from a phase I/II study exploring the combination of LT and ibrutinib in patients with DLBCL and mantle cell lymphoma demonstrated antitumor activity and manageable toxicity [126]. Current evaluations include its combination with rituximab in R/R follicular lymphoma and various DLBCL settings (NCT04998669, NCT05144009, NCT04384484).

#### Trastuzumab emtansine

In the phase III trials KAITLIN, MARIANNE, and KRISTINE, the combination of T-DM1 with pertuzumab, whether used in early or advanced HER2-positive BC, did not show improved clinical activity compared to the standard of care [127–129]. The phase II trial TEAL explored the combination of T-DM1, lapatinib, and nab-paclitaxel versus trastuzumab, pertuzumab, and paclitaxel in HER2-positive BC in the neoadjuvant setting. The experimental arm was associated with higher activity compared to the standard arm [130]. The phase III study HER2CLIMB-02 investigated the combination of T-DM1 and tucatinib in advanced HER2-positive BC, presenting results at the San Antonio Breast Cancer Symposium 2023. This combination significantly improved PFS compared to the control arm, also showing responses in patients with brain metastasis. However, it was associated with a higher rate of AEs, although generally manageable [131].

Neratinib, an irreversible panHER inhibitor, has the potential to overcome trastuzumab resistance by inhibiting downstream pathways [132]. In a small cohort of patients with HER2-positive mBC, the combination yielded an ORR of 63% with an acceptable safety profile [133]. Other studies are exploring the combination of T-DM1 with ribociclib or alpelisib in patients with HER2-positive mBC demonstrating good tolerability and promising activity [134, 135].

The combination of T-DM1 and pertuzumab was explored in the HERACLES-B trial for patients with HER2-positive advanced colorectal cancer, but the trial failed to meet its primary endpoint (ORR ≥ 30%) [136]. Another study investigating the combination of osimertinib plus T-DM1 in patients with advanced EGFR mutant and HER2-positive NSCLC exhibited limited efficacy [137].

#### Enfortumab vedotin

A phase I trial, evaluated EV with SG in mUC demonstrating significant clinical activity with the evidence of complete responses [138]. EV with erdafitinib is under evaluation in a phase I study involving patients with metastatic urothelial cancer (NCT04963153). Another trial is investigating EV in combination with cabozantinib in subjects with locally advanced or metastatic urothelial cancer (NCT04878029).

#### Sacituzumab govitecan

Preclinical evidence suggests a potential benefit of combining SG with polyadenosine-diphosphate-ribose polymerase (PARP) inhibitors in models of TNBC [139]. The combination of SG and rucaparib has been evaluated in the phase Ib SEASTAR study in patients with advanced TNBC, advanced platinum-resistant ovarian cancer, and solid tumors with mutations in homologous recombination repair genes. Despite signs of activity, further investigation is required due to safety concerns, particularly the high rate of myelosuppression [140]. Several studies are investigating SG plus talazoparib in metastatic TNBC (mTNBC) [141], and berzosertib [a potent and selective small-molecule Rad3-related kinase (ATR) inhibitor] in SCLC [142] and homologous recombination-deficient neoplasms who are progressive to PARP inhibitors (NCT04826341). Preliminary results from the phase I trial investigating SG and berzosertib have recently been published: objective responses were observed in 3 of 12 evaluable patients, and the ongoing phase II expansion cohorts are currently evaluating the efficacy [143].

#### Mirvetuximab soravtansine

The phase Ib/II FORWARD II evaluated MIRV, in combination with pembrolizumab and bevacizumab in patients with platinum-resistant ovarian cancer. The combination with bevacizumab is supported by evidence indicating enhanced antitumor activity, attributed to bevacizumab’s capacity to facilitate tumor penetration and exposure to the ADC [144]. It demonstrated notable effectiveness, yielding improved responses in patients regardless of their platinum sensitivity status. The combination was particularly effective in patients with high FRα expression tumors and in those who had not previously received bevacizumab [145]. These findings suggest that MIRV, in combination with bevacizumab, could represent a promising alternative to standard therapies for ovarian cancer, even for patients who have received prior treatments. A phase 1 study is currently assessing the combination of MIRV with rucaparib in patients with recurrent endometrial, ovarian, fallopian tube, or primary peritoneal cancer (NCT03552471). Another phase Ib trial is evaluating MIRV alongside SL-172154, a fusion protein consisting of human signal-regulatory protein alpha (SIRPα) and CD40L linked via a human Fc, in patients with platinum-resistant ovarian cancer (NCT05483933).

### Combinations of not-approved ADCs with targeted therapies

#### Belantamab mafodotin

The combination of BM with lenalidomide and dexamethasone has been evaluated in two clinical trials: the BelaRd study for naive MM patients and the DREAMM-6 study for R/R MM patients. Both studies indicated a rate of G3 AEs up to 94% across various dose levels. However, these AEs were generally manageable with dose modifications, and there were notable signs of clinical activity [146, 147]. The phase III trial DREAMM-8 is currently exploring BV with dexamethasone and pomalidomide in R/R MM (NCT04484623). Other ongoing combination regimens include BM with lenalidomide and daratumumab in relapsed or newly diagnosed MM (NCT04892264), BM plus bortezomib and dexamethasone (NCT04246047), among others.

#### Anetumab ravtansine

A phase II trial assessing the combination of AR with bevacizumab, compared to paclitaxel with bevacizumab in patients with platinum R/R ovarian cancer, reported poorer outcomes with the AR and bevacizumab combination, leading to the study’s termination [148].

#### Patritumab deruxtecan

Patritumab deruxtecan consists of an anti-HER3 mAb attached to a topoisomerase I inhibitor via a cleavable linker [149]. Preclinical findings showed that the therapy with EGFR-TKI increases HER3 expression, thus improving the anticancer activity of patritumab deruxtecan [150] and providing a rationale for an ongoing study which is evaluating patritumab deruxtecan plus osimertinib in patients with advanced EGFR-mutated NSCLC (NCT04676477).

#### Coltuximab ravtansine

Coltuximab ravtansine (SAR3419) is an ADC consisting of an anti-CD19 mAb conjugated with a cleavable linker to DM4 [151]. It has shown promising activity as a single agent in a phase II study in R/R DLBCL with benefits in PFS and OS [152]. A phase II trial was conducted in combination with rituximab in subjects with R/R DLBCL. The primary goal of ORR was not met, and there are no ongoing studies at the moment exploring this drug [153].

#### Moxetumomab pasudotox

Moxetumomab pasudotox (MOXE) is an ADC composed of a mAb anti-CD22 linked to pseudomonas exotoxin A (PE38). The drug received FDA approval in 2018 for the treatment of patients with pretreated hairy-cell leukemia (HCL) [154]. In July 2023 the company AstraZeneca decided to remove the drug from the market due to lack of use and the availability of other treatment options [155].

## Discussion and future directions

Over the last years there has been a significant increase in the number of ADCs entering preclinical and clinical development. In addition to the approved single-agent compounds, some of them have been approved also in combination with other anti-cancer agents, while many others are being tested in different combinations and phases of clinical development. Combination therapies have been considered as a possibility to increase the efficacy of ADCs. The most significant results have been achieved by combining ADCs with chemotherapy and more recently with ICIs. The approved combinations with chemotherapy have been developed in the field of hematological malignancies. As reported above, BV has been approved in combination with traditional chemotherapy in HL and T-cell lymphoma [23, 27], added to an established chemotherapy regimen by replacing one of the chemotherapy drugs (due to overlapping toxicity). While successful, combinations of ADCs with standard chemotherapy present also some challenges, in particular the definition of the correct dose and treatment schedule and thus setting a balance between toxicity and efficacy. In cases like the reapproval of GO for the treatment of AML with DA, lower fractionated dosing schedules were necessary [16]. The latest drug approved in combinations for hematologic malignancies is PV. This drug has not received approval as monotherapy but has been directly approved in combination with rituximab and bendamustine or R-CHP for the treatment of relapsed and treatment naive DLBCL respectively, the latter based on improvement only of PFS. Thus when combining ADCs with chemotherapy particular attention to toxicity and careful dose escalation schemes should be adopted. In addition, clear clinical benefits and superiority over the standard chemotherapy regimen should be demonstrated in randomized trials.

Beyond combinations with chemotherapy, preclinical evidence supporting the combination of ADCs with ICIs has prompted several clinical trials aimed at evaluating the safety and efficacy of such combinations. The combination of EV and pembrolizumab was approved by the FDA in December 2023 for the treatment of urothelial cancer, based on results from a phase III trial, which demonstrated improved OS [12]. Results from other combinations are awaited.

Despite their potent antitumor activity observed in different tumor types, the use of ADCs still presents several challenges, including safety but also patient selection, two factors that may become even more relevant when considering combination strategies. With regard to safety, there is a notable difference between ADCs and the naked mAb. ADCs have dose-limiting toxicities that are associated with the chemotherapy agents they are linked to, the composition of the ADC, and target expression in normal tissues. On the other hand, selecting those patients more likely to benefit remains largely an open question. Indeed despite required, antigen expression has not been clearly associated with antitumor activity in most of the cases [156, 157].

**Figure 2:**
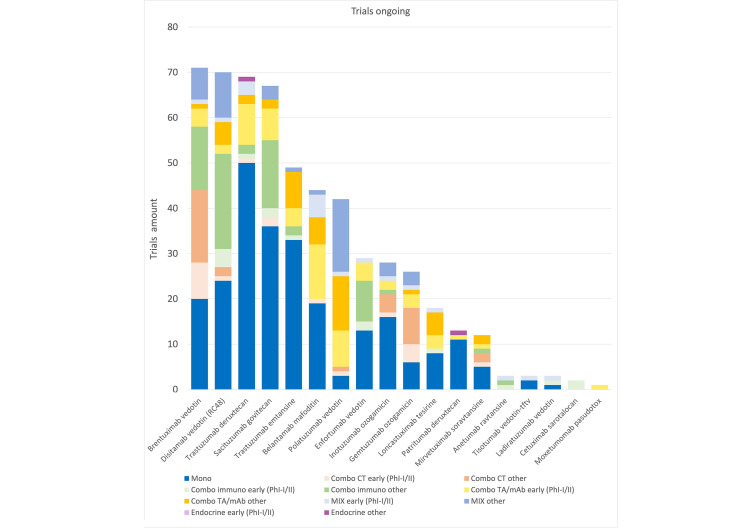
Trials ongoing. Graphic representation of ongoing studies: active not recruiting, recruiting, active not yet recruiting. Data obtained from ClinicalTrial.gov, updated as of January 2024. CT: chemotherapy; mAb: monoclonal antibody; Mono: monotherapy; Ph: phase; TA: targeted agent; MIX: combination of three or more drugs

Currently many trials are ongoing (Figure 2) that may better define in the near future the role of this class of compounds in the treatment of cancer and their incorporation in combination regimes. The emergence of novel constructs, such as bispecific ADCs, which allow simultaneous targeting of multiple antigens, potentially enhancing specificity and efficacy [158, 159], immunostimulatory antibody conjugates (ISACs) or immune checkpoint-targeted drug conjugates (IDCs), aiming to fuse the cytotoxicity of ADCs with immune-stimulatory properties, thereby amplifying the antitumor immune response [6, 160], may also open new possibilities for innovative strategies. Investigating predictive biomarkers and developing innovative preclinical models addressing the complexities of the tumor microenvironment could facilitate the translation of findings into clinically relevant strategies. Exploring these innovative modalities and their integration into combination strategies holds the potential to change the landscape of cancer therapy.

## Conclusions

Over the past decade, considerable progress has been made in the development of ADCs. A growing number of clinical trials are now exploring novel ADCs and their combinations with other therapies. Among these combinations, those involving chemotherapy were among the first to result in approvals for hematologic malignancies. However, they require special consideration due to associated toxicity. On the other hand, combinations with ICIs may present fewer overlapping toxicities. Future trials will need to address the optimal selection criteria for patients most likely to benefit from these combinations. Recently, the FDA approved the first combination of an ADC with an ICI for patients with urothelial cancer. Meanwhile, ongoing trials investigating combinations with small targeted agents and mAb across various tumor types have produced limited results thus far.

To ensure the successful development of treatment combinations based on ADCs in the future, it is crucial to establish preclinical rationale, conduct careful early clinical trials, and define clear efficacy endpoints for evaluation in phase II and III clinical trials.

## Abbreviations

ABVD: doxorubicin-bleomycin-vinblastine-dacarbazine
ADCs: antibody-drug conjugates
AEs: adverse events
ALL: acute lymphoblastic leukemia
AML: acute myeloid leukemia
AR: anetumab ravtansine
AVD: doxorubicin-vinblastine-dacarbazine
BC: breast cancer
BM: belantamab mafodotin
BV: brentuximab vedotin
CHOP: cyclophosphamide-doxorubicin-vincristine-prednisone
DA: daunorubicin and cytarabine
Dato-DXd: datopotamab deruxtecan
Depatux-M: depatuxizumab mafodotin
DLBCL: diffuse large B-cell lymphoma
EGFR: epidermal growth factor receptor
EV: enfortumab vedotin
FDA: Food and Drug Administration
FRα: folate receptor alpha
G3: grade 3
GO: gemtuzumab ozogamicin
HER2: human epidermal growth factor receptor 2
HL: Hodgkin lymphoma
ICIs: immune checkpoint inhibitors
INO: inotuzumab ozogamicin
la/mUC: locally advanced or metastatic urothelial carcinoma
LABC: locally advanced breast cancer
LT: loncastuximab tesirine
LV: ladiratuzumab vedotin
mAb: monoclonal antibody
mBC: metastatic breast cancer
MIRV: mirvetuximab soravtansine
MM: multiple myeloma
MMAE: monomethyl auristatin-E
NSCLC: non-small cell lung cancer
ORR: objective response rate
OS: overall survival
PD-L1: programmed cell death ligand 1
PFS: progression-free survival
PV: polatuzumab vedotin
R/R: relapsed or refractory
R-CHP: rituximab cyclophosphamide-doxorubicin-prednisone
SG: sacituzumab govitecan
T-DM1: trastuzumab emtansine
T-DXd: trastuzumab deruxtecan
TMZ: temozolomide
TNBC: triple-negative breast cancer

## Supplementary materials

The supplementary tables for this article are available at: https://www.explorationpub.com/uploads/Article/file/1002243_sup_1.pdf.

## Declarations

### Copyright

© The Author(s) 2024.
