# Supplementary material for: Antibody-drug conjugates combinations in cancer treatment
Source: Explor Target Antitumor Ther. 2024 Jun 27;5(3):714–41. doi: 10.37349/etat.2024.00243 (PMC11222717; doi:10.37349/etat.2024.00243)
Supplement: Supplementary file 1 [file 1002243_sup_1.pdf]

Table S1. Not-approved combinations of ADCs with chemotherapy with results

Year	Author	Combos	Comparator arm	Patient population	Ph	Efficacy	G 3-4 AEs
2016	Hutter-Kronke	GO + high dose cytarabine + mitoxantrone + all-trans retinoic acid	NA	R/R AML	II	ORR 61.5%	Septicemia 46%, pulmonary infection 22%
2023	Borchmann	BrECADD	eBEACOPP	Advanced stage HL	III	3-year PFS 94.9% vs. 92.3%	NK
2016	Ogura	INO + R-CVP	NA	R/R CD22 + B-cell non-HL	I	ORR at MTD 84%	Neutropenia 74%, thrombocytopenia 50%
2017	Shangs	INO + R-GDP	NA	R/R CD22 + B-cell non-HL	I	ORR 53%	85%
2023	Kantarjian	INO + mini-hyper-CVD –/+ blinatumumab	NA	pre-B ALL	II	ORR 76-93%; 3-year OS 34–52%	Most common: infections, hyperglycemia
2023	Jabbour	INO + mini-hyper-CVD	NA	pre-B ALL (older population)	II	2-year PFS 58.2%, 5-year PFS 44%	Trombocytopenia 78%, febrile neutropenia 32%
2016	Martin	T-DM1 + Docetaxel; T-DM1 + Docetaxel + Pertuzumab	NA	HER2 + mBC locally advanced BC	Ib	ORR 80.0% (mBC), pCR rate 60.3 (locally advanced BC)	Neutropenia 72% (mBC), 29% (locally advanced BC)
2016	Krop	T-DM1 + Paclitaxel + Pertuzumab	NA	HER2 + mBC	Ib/IIa	50%	77.3%
2020	Cortés	T-DM1 + Capecitabine	T-DM1	Previously treated HER2 + mBC	I/II	ORR 44% vs. 36%	44% vs. 41%
2022	Janjigian	T-DXd + 5FU (1); T-DXd + Capecitabine (2)	NA	HER2 + advanced or metastatic G/GEJ	Ib/II	NK	Anemia 33% (1) and 30% (2), neutropenia 33% (1) and

				cancer			40% (2)
2018	Moore	MIRV + carboplatin	NA	Platinum-sensitive relapsed ovarian cancer	Ib	ORR 71%, mPFS 15 m	Neutropenia 27.7%
2018	Bulat	AR + pegylated liposomal doxorubicin	NA	Platinum-resistant ovarian, fallopian tube, or primary peritoneal cancer	Ib	ORR 52%, DCR 86%	Neutropenia 23.8, thrombocytopenia 9.5%

AML: acute myeloid leukemia; AE: adverse event; AR: anetumab ravtansine; B-ALL: B acute lymphoblastic leukemia/lymphoma; BC: breast cancer; BrECADD: brentuximab vedotin, etoposide, cyclophosphamide, doxorubicin, dacarbazine, dexamethasone; CR: complete response; DCR: disease control rate; eBEACOPP: bleomycin, etoposide, doxorubicin, cyclophosphamide, vincristine, procarbazine, prednisone; EGFR: epidermal growth factor receptor; G/GEJ: gastric/gastroesophageal junction; GO: gemtuzumab ozogamicin; hyper-CVD: cyclophosphamide, vincristine, methotrexate, and cytarabine; HL: Hodgkin lymphoma; INO: inotuzumab ozogamicin; m: months; mBC: metastatic breast cancer; mOS: median overall survival; MIRV: mirvetuximab soravtansine; MTD: maximum tolerated dose; NA: not applicable; NK: not known; ORR: objective response rate; pCR: pathological complete response; PFS: progression free survival; R-CVP: rituximab, cyclophosphamide, vincristine, and prednisone; R-GDP: rituximab, gemcitabine, dexamethasone, and cisplatin; R/R: relapsed or refractory; SCLC: small cell lung cancer; T-DM1: Trastuzumab emtansine; T-DXd: Trastuzumab Deruxtecan; vs.: versus; +: positive

Table S2. Not-approved combinations of ADCs with ICIs with results

**Year**	**Author**	**Combos**	**Comparator arm**	**Patient population**	**Ph**	**Efficacy**	**G 3-4 AEs**
2019	Zinzani	BV + nivo	NA	R/R primary mediastinal B-cell lymphoma	I/II	ORR 71%, CR 37%	53%
2020	Diefenbach	BV + ipi; BV + nivo; BV + ipi and nivo	NA	R/R HL	I/II	ORR 76% vs. 89% vs. 82%	43% vs.16% vs. 50%
2020	Emens	T-DM1 + atezo	T-DM1 + placebo	HER2 + advanced BC	II	PFS 8.2 vs. 6.8 m	Thrombocytopenia 13% vs. 4%

2021	Hamilton	T-DM1 + atezo	NA	HER2 + locally advanced or mBC	Ib	ORR 35%	50-80%
2020	Waks	T-DM1 + pembro	NA	HER2 + mBC	Ib	ORR 20%, PFS 9.6 m	20%
2021	Hamilton	T-DXd + nivo	NA	HER2 + advanced breast and urothelial cancer	I	DCR 90.6% HER2 +, 75% HER2 low; PFS 8.6 m HER2 +, 6.3 m HER2 low	43.8%
2021	Schmid	T-DXd + durvalumab	NA	HER2 low advanced/ metastatic TNBC	Ib/II	ORR 100%	36%
2022	Grivas	SG + pembro	NA	mUC	II	ORR 34%	59%
2023	Jain	SG + ipi and nivo	NA	Cisplatin-ineligible mUC	I/II	ORR 66.6%	neutropenia (55.5%), anemia (33.3%)
2023	Vergote	TV + carboplatin 1L (arm D) pembro 1L (arm E) or 2L/3L (arm F)	NA	r/mCC	Ib/II	ORR: 54.5% arm D, 40.6% arm E, 35.3 % arm F	78.8% arm D, 66.7% arm E, 74.3% arm F
2018	Matulonis	MIRV + pembro	NA	FRα-expressing PROC	I/II	ORR 43% (MIRV + pembro)	NK
2020	Han	LV + pembro	NA	Advanced TNBC	I/II	ORR 54%	Neutropenia 16%
2022	Zhou	RC48-ADC + toripalimab	NA	Urothelial cancer	I/II	ORR 75%	Asthenia 8%
2024	Wang	RC48-ADC + toripalimab	NA	HER2 + G/GEJ cancer	I	ORR 43%, PFS 6.2 m, OS 16.8 m	59%
2022	Spiliopoulou	AR + nivo; AR + ipi/nivol; AR + nivo/gemcitabine	NA	Mesothelin-enriched advanced PDAC	I	DCR 22.2%, 25%, 100%	0%–19.5%

2023	Mansfield	AR + pembro	Pembro	Pleural Mesothelioma	I/II	PSF 12.2 vs. 3.9 m	NK
2021	Callander	BM + aICOS	NA	R/R multiple myeloma	I/II	ORR 48%	52%
2022	Suvannasankha	BM + pembro	NA	R/R multiple myeloma	I/II	ORR 47%, PFS 14.7 m	65%
2022	Levy	Dato-DXd + pembro + platinum based chemo	NA	Advanced NSCLC	Ib	ORR 39%, 69% (first line), DCR 82.6%	43%
2023	Schmid	Dato-DXd + dirvalumab	NA	First-line locally advanced/ metastatic TNBC	Ib/II	ORR 79%, median PFS 13.8 m	57%

Legend: AE: adverse event; aICOS: inducible costimulatory T-cell molecule agonist; AR: anetumab ravtansine; AST: aspartate aminotransferase; atezo: atezolizumab; BC: breast cancer; BM: Belantamab mafodotin; BV: Brentuximab Vedotin; CR: complete response; Dato-DXd: datopotumab deruxtecan; DCR: Disease Control Rate; G/GEJ: gastric or gastroesophageal junction; HL: Hodgkin lymphoma; Ipi: pipilimumab; LV: Ladiratuzumab vedotin; m: month; mBC: metastatic breast cancer; MIRV: mirvetuximab soravtansine; MOXE: moxetumomab pasudotox; mUC: metastatic urothelial carcinoma; NA: not applicable; nivo: nivolumab; NK: not known; NSCLC: non-small cell lung cancer; ORR: objective response rate; pembro: pembrolizumab; PDAC: pancrearic adenocarcinoma; PFS: progression free survival; PROC: platinum-resistant ovarian cancer; RC48-ADC: disitamab vedotin; r/mCC: recurrent or metastatic cervical cancer; R/R: relapsed or refractory; SAR3419: Coltuximab ravtansine; SG: sacituzumab govitecan; T-DM1: Trastuzumab emtansine; T-DXd: Trastuzumab Deruxtecan; TNBC: triple negative breast cancer; TV: Tisotumab vedotin; vs: versus; +: positive

Table S3. Not-approved combinations of ADCs with targeted therapy with results

**Year**	**Author**	**Combos**	**Comparator arm**	**Patient population**	**Ph**	**Efficacy**	**G 3-4 AEs**
2022	Bartlett	BV + lenalidomide + rituximab	NA	R/R DLBCL	III	ORR 70%	Most common: pneumonia and neutropenia
2022	Olszewski	PV + mosunetuzumab	NA	R/R DLBCL	Ib/II	ORR < 65 vs. ≥ 65 years: 54% vs. 72%	< 65 vs. ≥ 65 years: 39% vs. 58%
2019	Morschhauser	PV + rituximab	Pinatuzumab vedotin + rituximab	R/R DLBCL (1) or R/R FL (2)	II	ORR (1) 54% vs. 26%; (2) 70% vs.	(1) 77% vs. 79%; (2) 50% vs. 62%

						62%	
2020	Gritti	PV + venetoclax + rituximab	NA	R/R DLBCL	Ib/II	Median PFS 4.4 m; median OS 11.0 m	79%
2021	Bannerji	PV + obinutuzumab + venetoclax	NA	R/R FL	Ib/II	12m PFS 73%, median PFS NR	73%
2012	Ogura	INO + rituximab	NA	R/R CD22 positive B-cell non-Hodgkin lymphoma	I	ORR 80%	thrombocytopenia 70%, neutropenia 50%
2013	Fayad	INO + rituximab	NA	Non Hodgkin lymphoma	I/II	ORR FL 87%, DLBCL 74%	Thrombocytopenia 31%, neutropenia 22%
2014	Dang	INO + rituximab	Chemo of investigator's choice	R/R CD22 positive B-cell non-Hodgkin lymphoma	III	ORR 41% vs. 44%	NK
2022	Jain	INO + bosutinib	NA	R/R Ph+ ALL, LBP-CML	I/II	CR 83%	Thrombocytopenia 60%, neutropenia 38%
2022	Pirosa	INO + temsirolimus	NA	R/R CD22 positive B-cell non-Hodgkin lymphoma	I	PR FL 39%	Most common: thrombocytopenia and neutropenia
2020	Depaus	LT + ibrutinib	NA	R/R DLBCL or MCL	I/II	ORR 77.3%	41.2%
2022	Krop	T-DM1 + pertuzumab	Taxane + pertuzumab + trastuzumab	HER2 + early breast cancer	III	IDFS 93.1% vs. 94.2%	51.8% vs. 55.4%
2019	Perez	T-DM1 + pertuzumab	Trastuzumab + taxane	HER2 + advanced breast cancer	III	51.8% vs. 50.9%	48.6% vs. 55.8%
2019	Hurvitz	T-DM1 + pertuzumab	Docetaxel + carboplatin + trastuzumab +	HER2 + early breast cancer	III	3-year IDFS 93.0% vs. 92.0%	31.8% vs. 67.7%

			pertuzumab				
2019	Patel	T-DM1 + lapatinib + nab-paclitaxel	Trastuzumab + pertuzumab + paclitaxel	HER2 + early breast cancer	II	Residual cancer burden 0 or I: 100% vs. 62.5%	41% vs. 6%
2024	Hamilton	T-DM1 + tucatinib	T-DM1 + placebo	HER2 + mBC	III	Median PFS 9.5 vs. 7.4 m	Higher rate of hepatic events in combo arm
2019	Abraham	T-DM1 + neratinib	NA	HER2 + mBC	I/II	ORR 63%	Electrolyte abnormality 19%, thrombocytopenia 15%
2021	Spring	T-DM1 + ribociclib	NA	HER2 + mBC	Ib	Median PFS 10.4 m	neutropenia 33%, leukopenia 33%
2018	Jain	T-DM1 + alpelisib	NA	HER2 + mBC	I	ORR 43%, median PFS 8.1 m	59%
2020	Sartore-Bianchi	T-DM1 + pertuzumab	NA	HER2 + colorectal cancer	II	ORR 9.7%, median PFS 4.1 m	6%
2023	Jebbink	T-DM1 + osimertinib	NA	EGFR mutated NSCLC	I/II	ORR 4%, median PFS 2.8 m	14%
2024	McGregor	EV + SG	NA	mUC	I	ORR 70%	78%
2022	Yap	SG + rucaparib	NA	Cancer +/- mutations in homologous recombination repair genes	Ib	ORR 50%	83.3%
2023	Abel	SG + berzosertib	NA	Solid tumors	I	ORR 25%	NK
2023	Gilbert	MIRV + bevacizumab	NA	FRα-expressing PROC	Ib/II	ORR 44%	Hypertension 15%, AST increase 4%
2020	Popat	BM + bortezomib+ dexamethasone	NA	R/R MM	I/II	ORR 78%	89%
2022	Quach	BM + lenalidomide +	NA	R/R MM	I/II	ORR 42-75%	42–85%

		dexamethasone					
2023	Terpos	BM + lenalidomide + dexamethasone	NA	Transplant ineligible, newly diagnosed MM	I/II	ORR 100%	Fatigue 58%, rash 17%
2022	Lheureux	AR + bevacizumab	Paclitaxel + bevacizumab	PROC	I	ORR 18% vs. 55%; PFS 5.3 vs. 9.6 m	NK

Legend: AE: adverse event; AR: anetumab ravtansine; BM: Belantamab mafodotin; CR: complete response; DLBCL: diffuse large B-cell lymphoma; EV: Enfortumab vedotin; FL: follicular lymphoma; IDFS: invasive disease-free survival; INO: inotuzumab ozogamicin; LBP-CML: lymphoid blast phase of chronic myeloid leukemia; LT: Loncastuximab Tesirine; mBC: metastatic breast cancer; MCL: mantle cell lymphoma; MIRV: mirvetuximab soravtansine; MM: multiple myeloma; mUC:metastatic urothelial carcinoma; NA: not applicable; NK: not known; NR: not reached; NSCLC: non-small cell lung cancer; ORR: objective response rate; PFS: progression free survival; Ph+ ALL: philadelphia chromosome positive acute lymphoblastic leukemia; PR: partial response; PROC: platinum-resistant ovarian cancer; PV: polatuzumab vedotin; R/R: relapsed or refractory; SG: sacituzumab govitecan; T-DM1: trastuzumab emtansine; +: positive
